# Association of Corneal Biomechanics Properties with Myopia in a Child and a Parent Cohort: Hong Kong Children Eye Study

**DOI:** 10.3390/diagnostics11122357

**Published:** 2021-12-14

**Authors:** Shu-Min Tang, Xiu-Juan Zhang, Marco Yu, Yu-Meng Wang, Carol Y. Cheung, Ka-Wai Kam, Alvin L. Young, Li-Jia Chen, Clement C. Tham, Chi-Pui Pang, Jason C. Yam

**Affiliations:** 1 Department of Ophthalmology, The First Affiliated Hospital of Fujian Medical University, Fuzhou 350004, China; mushroomcave@163.com; 2 Department of Ophthalmology and Visual Sciences, The Chinese University of Hong Kong, Hong Kong 999077, China; zhangxiujuan@cuhk.edu.hk (X.-J.Z.); yumengwang@cuhk.edu.hk (Y.-M.W.); carolcheung@cuhk.edu.hk (C.Y.C.); kwkam@cuhk.edu.hk (K.-W.K.); b400128@cuhk.edu.hk (A.L.Y.); lijia_chen@cuhk.edu.hk (L.-J.C.); clemtham@cuhk.edu.hk (C.C.T.); cppang@cuhk.edu.hk (C.-P.P.); 3 Singapore Eye Research Institute, Singapore National Eye Centre, Singapore 168751, Singapore; marcocyyu@cuhk.edu.hk; 4 Department of Ophthalmology and Visual Sciences, Prince of Wales Hospital, Hong Kong 999077, China; 5 Hong Kong Hub of Paediatric Excellence, The Chinese University of Hong Kong, Hong Kong 999077, China; 6 Hong Kong Eye Hospital, Hong Kong 999077, China

**Keywords:** corneal biomechanics, myopia, axial length, children, adults

## Abstract

Associations between corneal biomechanics, axial elongation and myopia are important but previous results are conflicting. Our population-based study aimed to investigate factors associated with corneal biomechanics, and their relationships with myopia in children and adults. Data from 3643 children and 1994 parents showed that children had smaller deformation amplitudes (DA) than parents (*p* < 0.001). A larger DA was significantly associated with elongated axial length (AL; children: ß = 0.011; adults: ß = 0.0013), higher corneal curvature (children: ß = 0.0086; adults: ß = 0.0096), older age (children: ß = 0.010; adults: ß = 0.0013), and lower intraocular pressure (IOP; children: ß = −0.029; adults: ß = −0.031) in both cohorts. The coefficient of age for DA in children was larger than in adults (*p* < 0.001), indicating that the DA change with age in children is faster than in adults. DA was significantly associated with spherical equivalent (*p* < 0.001) resulting from its correlation with AL and corneal curvature. In conclusion, the cornea is more deformable in adults than in children, whereas corneal deformation amplitude increases faster with age in children than that in adults, along with AL elongation. Longer AL, steeper corneal curvature, older age and smaller IOP correspond to a more deformable cornea. The association between corneal deformation amplitude and refraction was mediated via AL and corneal curvature.

## 1. Introduction

Myopia is the most common ocular disorder worldwide, affecting as many as 90% of high school students in East Asia [1,2,3,4] Its prevalence in young adults is up to 80% in Korean military conscripts [5] and 70% in medical students in China [6] It is predicted that nearly half of the world’s population will be myopic by 2050, with as much as 10% being highly myopic [7,8] Understanding the pathogenesis of myopia development should help in finding preventive measures or remedies. In highly myopic eyes, progressive axial elongation results in a higher risk for vision-threatening complications, including glaucoma, retinal detachment, choroidal neovascularization, and myopic foveoschisis [1,2,3,9] This may be attributed to the eyeball’s biomechanical properties, that is, a more deformable eyeball leading to a larger axial elongation. Establishing this association is crucial and may shed the light on eyeball biomechanics as a parameter for myopia control and prevention of high myopia complications. 

Scleral biomechanics cannot be conveniently measured in vivo. Complex technologies with sophisticated software, including computerized tomographic (CT) scans and magnetic resonance imaging (MRI), have been used to investigate the biomechanical properties of the globe or sclera in live humans; however, both CT and MRI examinations are expensive, time-consuming, not widely available in eye clinics, and the acceptability of performing these exams in children is low. Ocular response analyzer (ORA), a non-contact tonometer, was proposed to assess corneal biomechanics in a more cost-effective and non-invasive way [10] Corneal hysteresis (CH) measured by ORA has been shown to correlate with corneal thickness [11,12] but varies over a wide range [11] In a Singaporean-children-based study, the CH and corneal resistance factor (CRF) have no association with refractive error and axial length (AL) [13] While a lower CH was associated with a higher degree of myopia [12] results of corneal biomechanics studies of ORA were inconsistent in these associations [14]. 

The advent of corneal visualization Scheimpflug technology, Corvis ST, allows direct real time visualization of corneal deformation response to an air pulse [15] Corneal deformation amplitude, a parameter measured by Corvis ST to quantify the maximum deformation amplitude of cornea from the start to the highest concavity of the cornea at the corneal apices with high repeatability and reproducibility [16,17,18] is a better way to assess the corneal elasticity compared with CH and CRF [17,19,20] Previous clinic-based studies have shown that corneal biomechanics measured by Corvis ST are associated with IOP, CCT, and age [21,22,23,24] 

Nevertheless, large-scale population-based studies to confirm the determinants for corneal biomechanics are lacking. Differences of corneal biomechanics in children and adults are unknown. Associations between corneal biomechanics, axial elongation and myopia are important but previous results are conflicting. We therefore conducted a population-based study of two Chinese cohorts: a child cohort, aged 6–8 years, and their parents, aged 25–70 years, with the following aims: (1) to describe the difference of corneal biomechanics properties, and their determinants in the two cohorts and (2) to fully evaluate the association between corneal biomechanics and myopia.

## 2. Materials and Methods

### 2.1. Participants and Study Design

The current study utilized a subgroup of the Hong Kong Children Eye Study (HKCES) cohort, which is a population-based study [25,26] Two cohorts were recruited at random from the children and parents. Exclusion criteria: subjects with significant systemic illnesses and ocular conditions, including congenital ocular disorders, media opacity, uveitis or a history of intraocular surgery, refractive surgery, glaucoma, or retinal diseases. This study followed the principles of the Declaration of Helsinki and was approved by the local research ethics committee, and informed consent was obtained. 

### 2.2. Measurement of Corneal Biomechanics

Corneal biomechanics measurements of both eyes of all the study subjects were conducted. Data obtained from the right eye were taken for statistical analysis. If the right eye was ineligible, data for the left eye were used. The corneal biomechanics parameters were documented by Corvis ST (OCULUS Optikgeräte GmbH, Wetzlar, Germany) for both the child and parent cohorts. After a constant air puff with a pressure of 60 mmHg, the cornea moved inwards until it reached maximum deformation and then moved back to its original position. Equipped with an ultra-high speed Scheimpflug camera (4330 frames/s), 140 images were acquired within 30 milliseconds by Corvis ST. On the display, images of the first and second applanation were documented. The first inward applanation occurred when an air puff was delivered to the eye, to flatten the cornea, whereas the second outward applanation was the flattened status of the cornea when it rebounded from its highest concavity. The corneal biomechanics parameters documented by Corvis ST included: inward corneal applanation lengths (A1L): length of the flattened cornea at the first applanation, in millimeters (mm); inward corneal applanation velocity (A1V): cornea velocity of apex at the first applanation, in meters/second (m/s); outward corneal applanation length (A2L): length of the flattened cornea at the first applanation, in mm; outward corneal applanation velocity (A2V): cornea velocity of apex at the second applanation, in m/s; deformation amplitude (DA): maximum deformation amplitude of cornea from start to the highest concavity of the cornea at the corneal apices, in mm; peak distance (PD): distance of the two apices of the cornea at the time of the highest concavity, in mm; and radius of curvature (RC): radius of curvature of a circle that fitted to corneal concavity at the time of the maximum deformation, in mm. DA was recognized to be the most direct parameter with high repeatability and reproducibility reflecting the elasticity of eyeball [16,17,27] Therefore, DA was adopted as a primary parameter of corneal biomechanics. 

### 2.3. Measurement of Refractive Error, Corneal Curvature and Axial Length

The refractive error and corneal curvature were measured using an auto-refraction/keratometer (ARK-530; Nidek, Gamagori, Japan) after cycloplegia. AL was measured by laser interferometry (IOL Master; Carl Zeiss Meditec, Jena, Germany). 

### 2.4. Statistical Analysis

**Figure 1:**
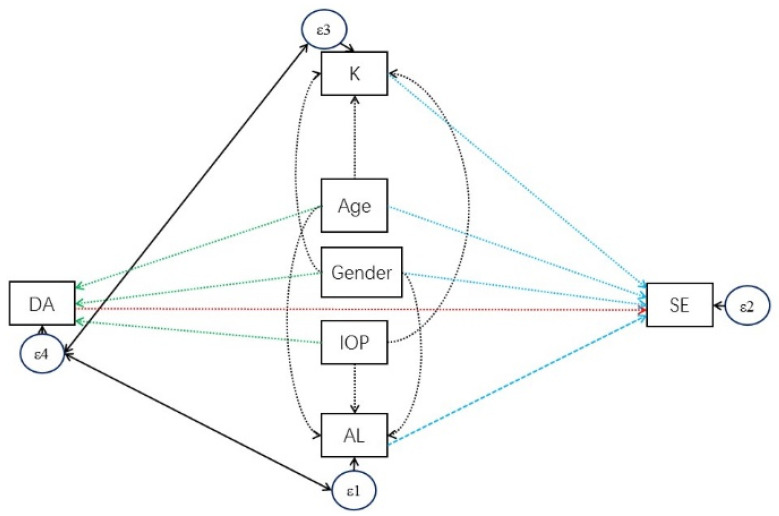
Structural equation model (SEM) to analyze the structural relationship between deformation amplitude (DA) and spherical equivalent (SE) after adjustment by the significant determinants found in multiple linear regression analysis. Directed arrows represent regression relationships; bidirected arrows represent association relationships. K: corneal curvature; AL: axial length.

**Figure 2:**
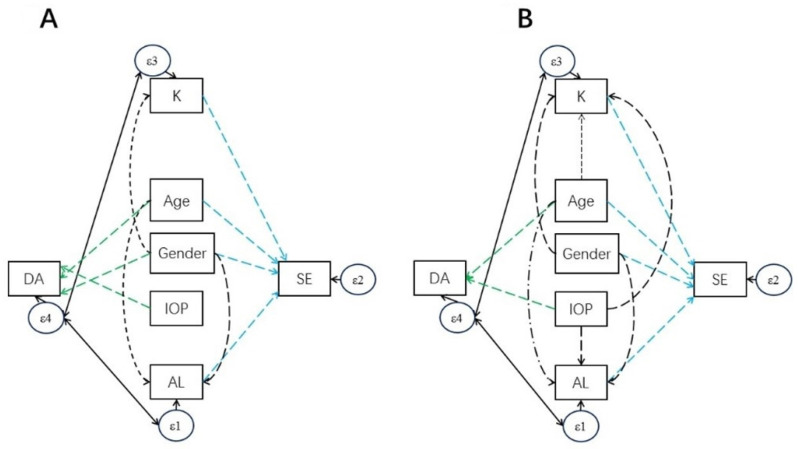
Final structural equation model (SEM) after backward selection approach. (**A**) final SEM in children; (**B**) final SEM in adults. Directed arrows represent regression relationships; bidirected arrows represent association relationships. K: corneal curvature; AL: axial length.

In this study, we examined the determinants of corneal biomechanics including DA, A1L, A2L, A1V, A2V, PD and RC, separately, for the child and parent cohorts, using univariate and multiple linear regression models. Among all corneal biomechanics parameters, DA was the primary outcome. Furthermore, to investigate whether the determinants for corneal biomechanics of children were different from adults, the coefficients of corneal biomechanics with determinants were compared between the children and the adults using Wald tests under linear mixed models adjusted for the parent–child correlation. Structural equation modeling is family of statistical methods for modeling relationship between variables; this was used in this study as a combination of factor analysis and multiple regression analysis, to evaluate the structural relationship between measured variables and latent constructs. We constructed a structural equation model (SEM) for DA and SE with incorporation of significant determinants found in multiple linear regression analysis (Figure 1), which can estimate the multiple and interrelated dependences in the model [28] SEM was then fitted for the child and parent cohorts separately. A backward selection approach was adopted to remove insignificant relations. The final models are presented in Figure 2. Analyses were performed using the statistical software STATA version 14 (StataCorp, College Station, TX, USA). A *p*-value < 0.05 was considered to be statistically significant. As a measure of the percentage of false positive results due to random error, false discovery rate (FDR) for multiple statistical tests with the threshold of *p*-value < 0.05 was evaluated for each table. It is estimated by $\frac{\left(number\;of\;tests\right)\times \left(the\;highest\;p-value\;obtained\;less\;than\;0.05\right)}{\left(number\;of\;significant\;discoveries\right)}\times 100\%$ [29].

## 3. Results

### 3.1. Characteristic of Children Cohort and Parent Cohort

We recruited 3643 eyes from 3643 subjects in the child cohort, and 1941 eyes from 1941 subjects in the adult cohort. We excluded 101 adults with either a history of intraocular surgery or suspected glaucoma. The means ± SD age of the child cohort and adult cohort were 7.66 ± 1.01 years and 41.09 ± 5.93 years, respectively. Table 1 summarizes the characteristics and the differences in corneal biomechanics between the two cohorts, and the descriptive statistics of the participants. All corneal biomechanics properties of children were significantly different from adults (Table 1).

### 3.2. Determinants for Corneal Biomechanics

The correlations between corneal biomechanics properties (DA, A1L, A2L, A1V, A2V, PD and RC) with AL, IOP, CCT, corneal curvature, age and gender in the child cohort, the parent cohort and the total cohort are summarized in Table 2. Increased DA, indicating a more deformable cornea, was significantly associated with elongated AL, lower IOP, higher corneal curvature and older age in both cohorts. No association between DA and CCT was found in the adults or children.

### 3.3. Comparison of the Coefficient of Determinants with DA between Two Cohorts

The coefficient of age with DA (ß coefficient: 0.011, *p*-value < 0.001, Table 2) in children was 7-fold larger than that in adults (ß coefficient: 0.0015, *p*-value < 0.001, Table 2), indicating the change of DA with age was faster in children than in adults (*p*-value < 0.001). Male gender was associated with a larger DA in children, but not in adults. Other coefficients of determinants with DA including AL, IOP, CCT, corneal curvature were similar between the two cohorts.

### 3.4. Analysis of the Association between SE and DA

**Figure 3:**
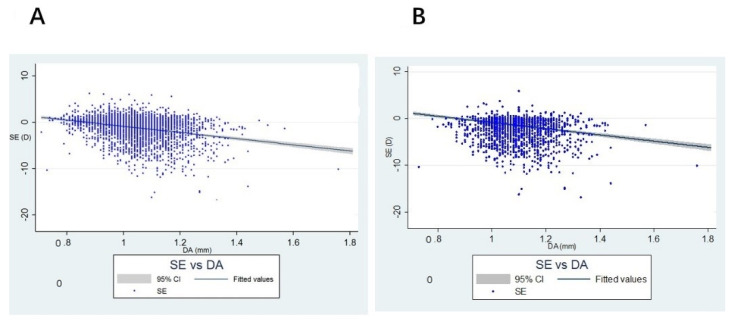
Scatter plot of the association between deformation amplitude (DA) and spherical equivalent (SE) in both children and adults. (**A**) scatter plot in children; (**B**) scatter plot in adults.

SE was significantly associated with DA in both children and adults (children: ß coefficient: −1.06 diopters per mm, *p*-value < 0.001, Figure 3A; adults: ß coefficient: −2.66 diopters per mm, *p*-value < 0.001, Figure 3B; simple linear regression (Table 3 and Table 4)). Based on the abovementioned multiple regression analyses for DA determinants, a SEM with DA as the exposure and SE as outcome was constructed (Figure 1). Insignificant relationships were removed and final models were constructed (Figure 2). DA became insignificant with SE in both children and parents. Positive correlations of 0.119 and 0.263 were obtained between DA and AL in children and adults, respectively, and positive correlations of 0.147 and 0.187 between DA and corneal curvature in children and adults, suggesting an indirect association between DA and SE mediated via AL and corneal curvature. We noticed that both AL and DA were associated with age in the SEM. Similar to the change of DA with age, the change of AL with age in children was 17-fold faster than that in adults (ß coefficients = 0.30 and 0.017, respectively). This pattern was not observed in the change of corneal curvature with age.

### 3.5. Correlation of Parental DA on Children’s DA

A total of 515 trios (father, mother, and child) with complete corneal biomechanic property measurements were included. Positive association of child’s DA with both the father’s (ß coefficient = 0.12, *p*-value = 0.006) and mother’s (ß coefficient = 0.15, *p*-value < 0.001) was shown in a simple linear regression, but not in multiple linear regression (father: ß coefficient = −0.019, *p*-value = 0.707; mother: ß coefficient = 0.045, *p*-value = 0.386).

## 4. Discussion

This study confirmed the correlation between myopia and corneal deformation amplitude in two Chinese cohorts: children and their parents. First, children have a less deformable cornea than adults. Boys have a marginally more deformable cornea than girls, but adults do not . In both children and adults, a more deformable cornea was associated with a longer AL, lower IOP, steeper corneal curvature and older age, but not with CCT. Second, we have identified a much larger increase (7-fold difference) in corneal deformation amplitude with age in children than in adults. This association shows the same trend to that of AL with age in both cohorts, and remained significant after adjustment with AL, suggesting a direct association between corneal biomechanics and age, independent of AL. Third, in the structural equation model (SEM), the associations between DA and SE were mediated via AL and corneal curvature. Fourth, the DA of children and of their parents were not correlated. 

A higher elasticity of cornea, indicated by increased DA, was associated with older age. In line with our findings, a similar age-related variation of corneal deformation in Japanese adults, aged 55.2 ± 16.1 years, was reported previously [22] Moreover, the corneal hysteresis and corneal resistance factor documented by ORA was decreased with age in 1136 Chinese adults [30] Previous studies have also shown men had lower corneal hysteresis than women [14,30] However, our study confirmed that corneal deformation is similar in men and women, although boys’ corneas may be mildly more deformable. In addition, regarding to CCT, we found it not associated with corneal deformation, but with other parameters including A1L, A1V and A2V. Corneal thickness does not affect the deformability of cornea, but it may affect the corneal dynamics and corneal resistance, in line with previous studies [30] 

Our unique inclusion of two large population-based cohorts, children and their parents, importantly, allowed us to determine the change of corneal biomechanics with age, which we found to be much faster in children than in adults. This may be explained by the ocular development of growing eyes in children with axial elongation. As expected, we also observed a much faster AL elongation in children than that in adults in our study, but other parameters, such as corneal curvature, remained stable with age. We confirmed the strong association between corneal biomechanics and myopic refraction, and we further constructed a SEM to analyze their relationship in details. Notably, this association was mediated via AL and corneal curvature. With stable corneal curvature with age, our result therefore suggested the change of corneal biomechanics with age is highly related to the change of axial elongation. It is, however, still unclear if an increase in DA is an effect or a consequence of an AL increase. Longitudinal follow up should confirm whether there is a causal relationship. 

Scleral remodeling based on changes in the composition of extracellular matrix altered the growth of the eyeball in animal studies [31,32] The properties of scleral extracellular matrix (including the collagen fibril, level of hydration, sclera fibroblasts) affect measurable outcomes of the scleral biomechanics [33] The scleral thinning in high myopia mammals corresponds to a general loss of collagen and proteoglycans [34] Sclera elasticity was increased during myopia development in animal models, and the alterations in scleral biomechanics in myopia were due to reduced collagen contents [33] However, the material properties and biomechanics of human sclera could be measured only in vitro [35] So far, there is no direct proof that the change of scleral properties was affected by the biomechanics of cornea. However, our study revealed the elongation of myopic eyes was associated with a more deformable cornea, suggesting the association of corneal and scleral biomechanics properties.

Our current finding of corneal biomechanics changes along with axial elongation is clinically relevant and important. It suggests a close relationship with refraction and axial length with corneal biomechanics properties, which may potentially be used to identify pre-myopic children at risk of developing myopia, and myopic children at risk of developing high myopia. Interestingly, the current study confirmed that cornea becomes more deformable with aging, and also with increased AL, which may shed light on the association between primary open angle glaucoma (POAG) and high myopia as well as aging. AL was found to be associated with POAG, and connective tissue changes with longer axial dimensions (AL) has been postulated as a potential mechanism. 

Our cohort is the largest in the literature on corneal biomechanics and myopia, involving more than 5000 individuals, both children and adults. Our findings represent population variations as our study subjects were recruited at random from a population-based study. Furthermore, we have identified the generation difference of corneal biomechanics properties in Chinese living in the urbanized environment of Hong Kong. Moreover, we adopted SEM in our analysis, which is a general modeling framework that incorporates many common statistical methods, including regression, analysis of variance (ANOVA), etc. SEM offers several advantages in our study. First, it allows for the estimation of multiple equations simultaneously, so that associations between multiple predictor and outcome variables can be assessed in the same model even when the distribution of outcome measures varies from dichotomous. Second, SEM provides a powerful tool for the assessment of mediation effects (i.e., AL and corneal curvature). Mediation is estimated and tested in a single step, with potentially more statistical power than traditional multistep methods. However, there were still some limitations of the current study. Firstly, a causative relationship between corneal biomechanics and axial elongation could not be concluded due to our cross-sectional design. The current study could only establish the relationship of axial length with corneal biomechanics. A longitudinal study should be warranted to further investigate the causative effect of axial elongation on corneal biomechanics. Secondly, results from Corvis ST can only represent the biomechanics of cornea but not the posterior part and not only the whole eyeball. We cannot extrapolate our findings to the total eyeball and retina layers, which means better non-invasive instruments should be invented to investigate the posterior biomechanics in future. Thirdly, it is well known in the statistical literature that analyzing data from a single eye is not free from bias. Therefore, from this point of view, the design of the research should optimally include measurements from both eyes. In current study, data from the right eyes were prioritized for analyses, which may lead to selection bias. It was because that many children were not cooperative when examined the second eye due to air puff. Usually, the quality of data from the right eyes were better and we found the data from both eyes were highly correlated.

In conclusion, cornea is more deformable in adults than in children, whereas corneal deformation amplitude increases faster with age in children than that in adults, along with AL elongation. Longer AL, steeper corneal curvature, older age and smaller IOP correspond to a more deformable cornea. The association between corneal deformation amplitude and refraction was mediated via AL and corneal curvature.

## Figures and Tables

**Table 1:** Characteristic of the study population.

	Total (*n* = 5584)		Children (*n* = 3643)		Adults (*n* = 1941)		*p* Value
	Mean ± SD	Range	Mean ± SD	Range	Mean ± SD	Range	
Deformation Amplitude (mm)	1.04 ± 0.12	0.51–2.5	1.01 ± 0.11	0.51–2.5	1.10 ± 0.10	0.73–1.76	<0.001
Applanation inwards (mm)	1.79 ± 0.14	0.49–3.91	1.81 ± 0.15	0.49–3.91	1.77 ± 0.11	1.21–2.79	<0.001
Velocity inwards (m/s)	0.17 ± 0.02	0.02–0.8	0.16 ± 0.02	0.02–0.8	0.17 ± 0.02	0.03–0.7	0.003
Applanation outwards (mm)	1.61 ± 0.42	0.24–3.63	1.59 ± 0.44	0.24–3.63	1.66 ± 0.37	0.35–2.79	<0.001
Velocity outwards (m/s)	−0.37 ± 0.11	−1.26–−0.01	−0.35 ± 0.12	−1.26–−0.01	−0.41 ± 0.09	−0.92–−0.09	<0.001
Peak distance (mm)	4.18 ± 1.13	0.62–9.7	4.2 ± 1.07	0.62–9.7	4.14 ± 1.24	0.89–8.63	0.047
Radius of curvature (mm)	7.1 ± 1.53	0.52–13.81	6.95 ± 1.65	0.52–12.02	7.38 ± 1.23	1.53–13.81	<0.001
IOP (mmHg)	15.45 ± 2.49	8–34.5	15.56 ± 2.59	8–34.5	15.26 ± 2.28	8–31	<0.001
CCT (μm)	547.08 ± 33.05	404–679	549.86 ± 32.47	404–679	541.84 ± 33.5	416–661	<0.001
Corneal curvature (Diopter)	43.6 ± 1.45	38.04–50.62	43.53 ± 1.41	38.04–49.27	43.73 ± 1.51	38.34–50.62	<0.001
Age (year)	19.87 ± 16.51	4.41–72.49	7.66 ± 1.01	4.41–11.36	41.09 ± 5.93	24.24–72.49	<0.001
AL (mm)	23.69 ± 1.39	19.55–34.94	23.17 ± 0.95	19.55–27.99	24.67 ± 1.55	21.14–34.94	<0.001
SE	−0.99 ± 2.76	−23.5–8.25	0.12 ± 1.60	−11–8.25	−3.08 ± 3.23	−23.5–5.88	<0.001
Number of myopia (*n*, %)	2486 (44.52%)		926 (25.41%)		1560 (80.50%)		<0.001

False discovery rate ≤4.7% is expected for the 12 significant results out of the 14 tests.

**Table 2:** Determinants of corneal biomechanics (linear mixed model).

	Corneal Biomechanics	Deformation Amplitude (mm)		Applanation Inwards (mm)		Velocity Inwards (m/s)		Applanation Outwards (mm)		Velocity Outwards (m/s)		Peak Distance (mm)		Radius of Curvature (mm)	
	Independent variables	ß	*p* value	ß	*p* value	ß	*p* value	ß	*p* value	ß	*p* value	ß	*p* value		
Children	AL	0.011	<0.001	−0.0017	0.647	7.52 × 10^−4^	0.11	−0.0023	0.818	−0.019	<0.001	0.026	0.30	0.031	0.40
	IOP	−0.029	<0.001	0.0070	<0.001	−0.0053	<0.001	0.0027	0.389	0.015	<0.001	−0.070	<0.001	0.059	<0.001
	CCT	−4.16 × 10^−5^	0.40	3.01 × 10^−4^	<0.001	8.48 × 10^−6^	0.44	0.0019	<0.001	2.67 × 10^−4^	<0.001	−5.73 × 10^−4^	0.33	0.0059	<0.001
	Corneal curvature	0.0086	<0.001	−0.018	<0.001	0.0025	<0.001	−0.012	0.060	−0.0073	<0.001	−0.0005	0.98	−0.13	<0.001
	Age	0.010	<0.001	−0.0036	0.202	−3.0 × 10^−4^	0.44	0.0057	0.470	−0.0053	0.007	−0.0017	0.93	0.092	1.30 × 10^−3^
	Gender	−0.0082	0.011	0.0041	0.471	0.0015	0.040	0.032	0.040	0.0026	0.49	−0.025	0.51	0.16	4.70 × 10^−3^
Adults	AL	0.013	<0.001	−1.54 × 10^−4^	0.927	0.00089	0.004	−0.022	<0.001	−0.013	<0.001	−0.004	0.83	−0.078	<0.001
	IOP	−0.031	<0.001	0.0060	<0.001	−0.0051	<0.001	0.013	<0.001	0.018	<0.001	−0.043	0.002	0.11	<0.001
	CCT	−7.82 × 10^−5^	0.15	6.02 × 10^−4^	<0.001	1.53 × 10^−5^	0.28	0.0023	<0.001	4.71 × 10^−4^	<0.001	1.48 × 10^−4^	0.87	6.98 × 10^−3^	<0.001
	Corneal curvature	0.0096	<0.001	−0.011	<0.001	3.07 × 10^−3^	<0.001	−0.026	<0.001	−6.72 × 10^−3^	<0.001	−1.93 × 10^−3^	0.92	−0.12	<0.001
	Age	0.0013	<0.001	−1.13 × 10^−5^	0.98	−0.00039	<0.001	0.0016	0.28	1.20 × 10^−5^	0.97	0.0037	0.48	8.13 × 10^-3^	0.093
	Gender	0.0040	0.28	−6.25 × 10^−4^	0.91	8.86 × 10^−4^	0.36	0.030	0.10	0.0048	0.25	−0.072	0.26	0.10	0.092
		*p* value		*p* value		*p* value		*p* value		*p* value		*p* value		*p* value	
Two Cohorts Coefficient Comparison	AL	0.092		0.031		0.70		0.26		0.75		0.13		0.002	
	IOP	0.19		0.77		0.40		0.073		0.005		0.064		0.013	
	CCT	0.62		0.13		0.90		0.089		0.19		0.49		0.24	
	Corneal curvature	0.29		0.99		0.25		0.43		<0.001		0.75		0.037	
	Age	<0.001		0.078		0.94		0.36		0.001		0.94		0.002	
	Gender	0.016		0.37		0.58		0.95		0.98		0.48		0.53	

False discovery rate ≤9.0% is expected for the 56 significant results out of the 126 tests. AL: axial length, IOP: intraocular pressure, CCT: central cornea thickness; ß: beta coefficient.

**Table 3:** Association of SE with corneal deformation amplitude in children (SEM).

Dependent Variables	Independent Variables	Coef.	95% CI		*p* Value
SE < --	K_average	−0.72	−0.7	−0.7	<0.001
	AL	−1.87	−1.9	−1.8	<0.001
	age	0.13	0.11	0.16	<0.001
	gender	−0.4	−0.5	−0.4	<0.001
	_cons	74.16	72.7	75.6	<0.001
DA < --	age	0.01	0.01	0.02	<0.001
	gender	−0.01	−0	0	0.006
	IOP	−0.03	−0	−0	<0.001
	_cons	1.37	1.34	1.4	<0.001
AL < --	age	0.30	0.27	0.32	<0.001
	gender	−0.60	−0.7	−0.5	<0.001
	_cons	21.81	21.6	22.1	<0.001
K < --	gender	0.69	0.6	0.79	<0.001
	_cons	42.48	42.3	42.6	<0.001
	var(e.DA)	0.01	0.01	0.01	
	var(e.k_average)	1.86	1.77	1.95	
	var(e.SE)	0.51	0.49	0.54	
	var(e.AL)	0.71	0.68	0.75	
cov(e.DA,e.K)		0.02	0.01	0.02	<0.001
cov(e.DA,e.AL)		0.01	0.01	0.01	<0.001

SE: spherical equivalent, DA: deformation amplitude, AL: axial length, K: corneal curvature.

**Table 4:** Association of SE with corneal deformation amplitude in adults (SEM).

Dependent Variables	Independent Variables	Coef.	95% CI		*p* Value
SE < --	K_average	−0.79	−0.84	−0.74	0.004
	AL	−1.97	−2.02	−1.93	<0.001
	age	0.033	0.021	0.045	<0.001
	gender	−0.70	−0.85	−0.55	<0.001
	_cons	79.91	77.28	82.54	<0.001
DA < --	age	0.002	0.001	0.002	<0.001
	IOP	−0.030	−0.031	−0.028	<0.001
	_cons	1.49	1.46	1.52	<0.001
AL < --	age	0.017	0.005	0.029	0.006
	gender	−0.57	−0.709	−0.423	<0.001
	IOP	0.047	0.018	0.077	0.002
	_cons	24.122	23.358	24.885	<0.001
K < --	age	0.018	0.006	0.030	0.004
	gender	0.591	0.448	0.733	<0.001
	IOP	0.068	0.039	0.097	<0.001
	_cons	41.055	40.304	41.807	<0.001
	var(e.DA)	0.006	0.006	0.006	
	var(e.K)	2.191	2.056	2.335	
	var(e.SE)	2.199	2.064	2.343	
	var(e.AL)	2.284	2.143	2.433	
cov(e.DA,e.K)		0.021	0.016	0.027	<0.001
cov(e.DA,e.AL)		0.031	0.025	0.036	<0.001

SE: spherical equivalent, DA: deformation amplitude, AL: axial length, K: corneal curvature.

## Data Availability

The data presented in this study are available on request from the corresponding author. The data are not publicly available due to privacy.
